# Revival of Mucin-Degrading Human-Derived Fecal Microbial Consortia After Cryopreservation

**DOI:** 10.17912/micropub.biology.002071

**Published:** 2026-06-08

**Authors:** Ava N. Tafreshi, Teanna Samuel, Gilberto E. Flores, Ashwana D. Fricker

**Affiliations:** 1 College of Arts and Sciences, Cornell University, Ithaca, NY, US; 2 Biology Department, Adelphi University, Garden City, NY, US; 3 Department of Biology, California State University, Northridge, Northridge, CA, US

## Abstract

Human gut microbial communities capable of degrading mucin are taxonomically unique and have a range of physiologically relevant metabolic outputs. To determine the feasibility of reviving mucin-degrading fecal microbial communities after cryopreservation, we employed 16S rRNA gene sequencing to characterize revived communities. Microbial communities were generally stable but small donor-dependent shifts in diversity, composition, and taxonomy were observed following revival. The revivability of these microbial communities is valuable for studying mucin-degrading microbial communities.

**Figure 1. After revival, microbial communities have small donor-dependent shifts in density, metabolic products, compositional diversity, and taxonomy:** A and B: There is a donor-dependent shift in both the OD _600_ (A) and pH (B) during revival as compared to the final developed community (striped bars). Averages of three lineages from each donor community are displayed with error bars representing standard deviation as calculated in R. C: Revived communities are similar in composition to the originally developed communities. Principal coordinates analysis plots of Bray–Curtis dissimilarity between communities for each donor comparing the final day of development and the 5 days of revival (PERMANOVA comparing days p>0.01 for all donors). Donors are represented by fill color, days by hue of the dominant color, and development or revival by shape. D: Dominant phyla are retained after cryopreservation and revival. The relative abundance of community members for each donor at day 10 of initial community development and across 5 days is averaged across three replicate lineages. The top fifty taxa across all donor communities are shown, where each shade represents a genus, the dominant color represents a phylum and the remaining low-abundance ASVs are grouped in Other (light grey). E: Species richness had donor-dependent shifts but plateaued after revival. Mean, first, and third quartiles of the number of ASVs are represented with fill color indicating donor. F: Taxa whose abundances were significantly different between the final days of development and revival had donor-dependent trends. Statistically significant differences were calculated by MaAsLin2 and comparisons were considered significant if the corrected p-values were less than 0.05. Only taxa whose abundances were significantly different in two or more communities are shown. Color palette ranging from cornsilk (low abundance) to blue (high abundance) represent abundances between the minimum and maximum values. G: *Escherichia-Shigella* and *Blautia* have inverse but consistent trends across communities. Averages of three replicate lineages from each donor community are displayed with error bars representing standard deviation as calculated in R. For all figures, communities are labeled based on the parent source (Donor 1 communities, D1; Donor 2 communities, D2; Donor 3 communities, D3). Except where indicated, statistically significant differences are calculated by Tukey’s multiple comparisons test with P<0.05. Symbol style: 0.05 (*), 0.01 (**), 0.001(***) and <0.0001(****), non significant (ns) comparisons not shown.

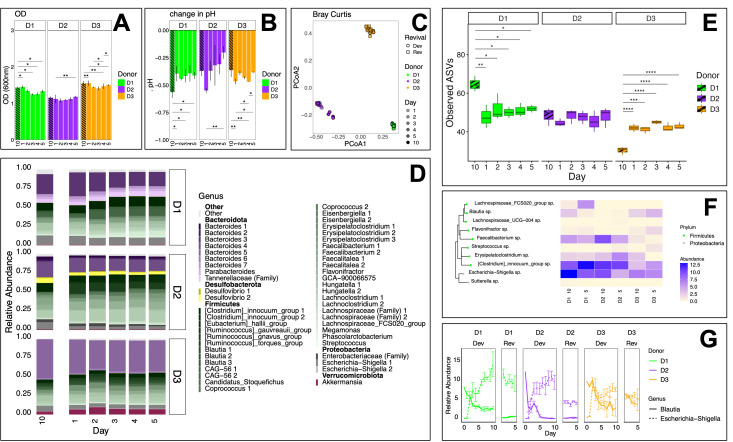



## Description

The microorganisms residing within the gut, collectively known as the gut microbiota, contribute to human health and disease (Fan & Oluf, 2021; Pascale et al., 2018). Every individual has a unique set of microorganisms that is shaped throughout their lifetime by several factors including diet (Mohammadkhah et al., 2018). Beyond diet, a group of bacteria of the gastrointestinal (GI) tract obtain carbon and nitrogen from mucins, a family of glycoproteins secreted by the host that form the main structural components of the protective mucus barrier (Leeming et al., 2019; Tailford et al., 2015). During consumption of mucins by some bacteria, metabolites such as acetate stimulate additional mucin production by epithelial cells, creating a positive feedback loop and resulting in an intact mucus barrier (Adamberg et al., 2018; Willemsen et al., 2003). This mucus barrier prevents infection by reducing the ability of pathogens to attach to epithelial cells (McGuckin et al., 2011). While a subset of microorganisms capable of mucin degradation has been identified, the full suite of mucin-fermenting microorganisms remains a mystery.

Previously, we showed that three mucin-degrading communities from humans are taxonomically unique yet functionally redundant, resulting in production of varying levels of short chain fatty acids (Fricker et al., 2024). These communities reflect a breadth of gut bacterial diversity and can be monitored through time. Future work aims to use these communities to unravel mechanistic interactions between organisms that are impacted by the strain-level diversity of the human gut microbiome. However, microbial community enrichments with specific assemblages are difficult to store, as each microorganism in the community responds differently to long-term storage conditions (Biclot et al., 2022). Therefore, we set out to determine the feasibility of reviving the three previously generated mucin-degrading fecal communities (Donor 1 communities, D1; Donor 2 communities, D2; Donor 3 communities, D3) after cryopreservation in 25% glycerol at -80 ^o^ C for >6 months. We hypothesized that some communities would demonstrate higher resilience to a freeze-thaw perturbation due to the specific taxonomic composition.

Communities generated previously and cryopreserved for >6 months were revived and subcultured daily for 5 days to determine the impact of storage on mucin community composition. After incubation of each community for 24h on mucin media, optical density (OD) and pH were determined as primary growth and metabolic outputs, respectively. A high OD (>1) across all donors indicates robust growth throughout the incubation period (Panel A). Comparing the OD of revived mucin-degrading communities across a 5-day period to the final developed community from which it was revived (day 10), revealed donor-dependent differences. The OD of D1 started high and then dropped marginally, but significantly, a trend which was mirrored by D3. However, the OD of D2 did not fluctuate over the revival period. By the end of the 5-day revival period, only D1 OD was significantly different from the community from which it was revived (day 10). Similar to observations of OD, more significant changes in pH were seen in D1 and D3, although patterns of pH changes were different across donors (Panel B). Comparing the pH on day 5 of revival to day 10 of development, only D1 produced significantly less acid, whereas the other two communities had nonsignificant but less acid production (D2) or no difference (D3). The acid is likely composed of short chain fatty acids (SCFAs), such as acetate, butyrate, and propionate from mucin glycan fermentation (Fricker et al., 2024). The differences in abundance of SCFAs is likely to be driven by specific cross-feeding interactions within each community, where, for example, *A. muciniphila* liberates sugars during mucin fermentation leading to increased butyrate production by *Eubacterium hallii* when in co-culture (Belzer et al., 2017).

To identify differences in microbial community composition, taxonomy, and diversity after cryopreservation, 16S rRNA gene sequences of revived communities were compared to sequences from the final time point of the original 10-day development. Compositionally, communities clustered by donor, suggesting that community composition is retained before and after freezing (Panel C). No shift in the composition of D1 and D3 were observed (PERMANOVA, p>0.05) and a non-significant but perceptible shift in the composition of D2 was observed (PERMANOVA, p>0.01).

Taxonomic compositions of the revived communities revealed that Bacteroidetes (Bacteroidota), Firmicutes (Bacillota), and Proteobacteria (Pseudomonadota) were abundant across all donors, and unique phyla to each donor included Desulfobacterota in D2 and Verrucomicrobiota in D3 (Panel D). These dominant taxa are in line with the original development of these communities (Fricker et al., 2024).

To determine impacts of cryopreservation on diversity, the number of observed ASVs in the communities before and after freezing were compared. Surprisingly, when comparing each day of revival to the final day of development, each community had a different pattern in which D1 had a reduction in ASVs (net loss of 21 ASVs), D2 had no significant changes, and D3 had an increase in ASVs (net gain of 24 ASVs) (Panel E). However, across both D1 and D3 communities, half of these sequences were present only once across all samples, and likely represent sequencing artifacts. Cumulatively, all of the lost ASVs from D1 represented less than 2.2% and gained ASVs from D3 represented less than 1.2% of the corresponding communities. Seven ASVs in the D3 samples were present at some point during the initial development of the consortia, suggesting they were present throughout development with fluctuating relative abundances that sometimes fell below the detection limit of sequencing. Of the remaining five ASVs in the D3 samples, none were present in all three replicate lineages consistently across the revival period nor in the initial development of the consortia, suggesting possible cross-contamination from other samples on the sequencing run. Further supporting this is the fact that all five ASVs are common gut microorganisms.

In order to identify taxonomic differences after revival, ASVs were collapsed to genera, and relative abundances from the end of the revival period (day 5) were compared to the final day of development (day 10) for each donor using MaAsLin 2. Then, genera significantly different in two or more donors were identified, revealing ten genera (Panel F). Of the ten genera that had significant changes in abundance between development and revival, three taxa, *Lachnospiraceae* UCG004, *Sutterella* , and *Streptococcus* , had abundances that fluctuated between 0 % - 1.6 %. Since the community structures also appeared very similar across day 10 of development and day 5 of revival (Panels C and D), we conclude that shifts in the abundance of these organisms does not exert a strong influence on the overall community structure. Three genera had the same trend across all donors, *Flavonifractor* increased after revival whereas both *Escherichia* - *Shigella* and *Faecalibacterium* decreased after revival. Some notable differences across donors were also observed. For instance, the relative abundance of *Erysipelatoclostridium* increased after revival as compared to development in D2 but decreased in D3 and was unaffected in D1.

To begin to assess potential interactions between community members, we observed the relative abundances of the aforementioned ten genera across both the initial development and subsequent revival. This revealed that two genera, *Escherichia-Shigella* and *Blautia* had an inverse relationship (Panel G). Namely, that the relative abundance of *Escherichia-Shigella* increased whereas the abundance of *Blautia* decreased during the initial community development. After thawing, the relative abundances of both *Escherichia-Shigella* and *Blautia* in the revived communities were consistent across each day of revival. First, this suggests that freezing and thawing retains the relationship between taxa within diversely populated microbiota. Second, the inverse abundance pattern observed here aligns with previous co-culture studies which find that *Blautia* has an inhibitory effect on *E. coli* growth (Wei et al., 2025). Further co-culture studies using isolates from these donors can unravel mechanistic interactions between organisms and determine the impact of the strain-level diversity across the human gut microbiome.

Here, we report on the composition of three revived unique fecal microbial communities after cryopreservation. The aim of this study was to determine the feasibility of future use of these mucin-degrading communities as model systems to study host-microbe, drug-microbe, and microbe-microbe interactions. Our findings suggest that the communities are generally stable after revival with minor shifts in the relative abundance of specific taxa. However, it is unclear whether these changes to community structure or how other types of perturbations, such as dietary glycans or xenobiotic compounds, influence mucin metabolism, which may be tested in future work. One final consideration is that repeat freeze-thaw cycles may impact evolution of individual taxa within the communities, as has been observed for individual species (Sleight et al., 2006). Therefore, careful monitoring of these communities through metagenomic analysis, limited subculturing, and long-term cryopreservation will limit the evolution of individual community members so that they can be used in future experiments aimed at understanding microbial interactions in the human gut microbiome.

## Methods

*Medium*

Preparation of fermentation media used for revival of communities was described previously for initial community development (Fricker et al. 2024).

To make fermentation media, concentrated fermentation buffer (2X) was prepared aerobically and diluted to a working concentration with sterile water and 0.5% filter sterilized (0.22 μM Whatman filters, Whatman, USA) soluble porcine gastric mucin. Porcine gastric mucin type III (Sigma Aldrich, USA) was prepared as previously described (Fricker et al. 2024; Kirmiz et al. 2020). Prior to transfer to Balch tubes, fermentation media was stored covered in the anaerobic chamber (85% N _2_ , 10% CO _2_ , 5% H _2_ ) for 48h to outgas. Each tube received 5 mL fermentation media. Fermentation media was aseptically distributed into Balch tubes in a 10% CO _2_ , 5% H _2_ , and 85% N _2_ atmosphere before sealing with butyl rubber stoppers. Immediately before inoculation, 0.05 mL ATCC vitamin mix [final 1% v/v, ATCC MD-VS, Hampton, NH] was added to each tube. An additional three tubes were maintained for the full duration of the experiment at 37 ^o^ C without inoculation as negative controls.

Final fermentation media contained 24 μM Na _2_ HPO _4_ , 15.6 μM NaH _2_ PO _4_ , 8 mM NaCl, 6 mM KCl, 6.7 mM NH _4_ Cl, 0.7 mM Na _2_ SO _4_ , 1 μg/mL Resazurin, 3.2 mM Urea, 1.4 mM Cysteine-HCl, 0.5 mM CaCl _2_ -2H _2_ O, 0.5 mM MgCl _2_ -6H _2_ O, 1.3 μM Na _2_ -EDTA -2H _2_ O, 0.6 μM CoCl _2_ -6H _2_ O, 0.5 μM MnCl _2_ -4H _2_ O, 0.36 μM FeSO _4_ -7H _2_ O, 0.7 μM ZnCl _2_ , 0.17 μM AlCl _3_ -6H _2_ O, 0.09 μM Na _2_ WO _4_ -2H _2_ O, 0.12 μM CuCl _2_ -2H _2_ O, 0.076 μM NiSO _4_ -6H _2_ O, 0.077 μM H _2_ SeO _3_ , 0.16 μM H _3_ BO _3_ , 0.04 μM NaMoO _4_ -2H _2_ O, 10 μM equimolar amino acid mix, and 0.5% soluble porcine gastric mucin type III.

*Inoculum Preparation and Community Revival*

During community development, the final communities (Day 10) were stored in 25% glycerol at -80 ^o^ C for long-term storage (Fricker et al. 2024). After storage for > 6 months (9 months for Donor 1 and Donor 2, 7 months for Donor 3), communities were revived as follows. Freezer stocks were thawed anaerobically at room temperature for 5-10 minutes. To remove glycerol, cultures were washed three times with fermentation media. Briefly, 750 μL of the thawed freezer stock was transferred to a microfuge tube and centrifuged at maximum speed (14,100 x g) for 2 minutes and the supernatant carefully removed by pipetting. Pellets were resuspended in a 1.5 mL fresh fermentation media by vortexing for 5 seconds at 3,000 RPM. The process of centrifugation and resuspension was repeated. After a second resuspension, microfuge tubes were centrifuged once more at 14,000 x g and resuspended in 600 μL fresh fermentation media by vortexing at 3,000 RPM for 5 seconds with additional 2 second pulses until the pellet visibly dissipated. Each replicate tube containing 5 mL of fermentation media received 200 μl of the resuspended pellet.

*IRB Approval*

The protocol for human fecal collection was approved by the Institutional Review Board of California State University, Northridge (#1516–146-f). No additional subjects were recruited to this study and all human-derived microbial communities were sourced from previously collected and cryopreserved samples. The study complied with all institutional and federal policies regarding the use of human subjects.

*In vitro sequential transfer*

The sequential cultivation experiment was continued for 5 consecutive days, and each donor was cultured in triplicate lineages that were not intermixed (Fricker et al. 2024; Yao, Chen, and Lindemann 2020). Daily, immediately prior to inoculation, ATCC vitamins was added to each tube of fermentation media as described above. Due to settling of microorganisms from static incubation, tubes were gently inverted 5x prior to sampling. Subsequently, each culture was subcultured as follows; 50 μL of the incubated culture was transferred to the corresponding tube containing a 5mL fermentation media and incubated in the Coy chamber as described above.

Daily, 500 μL of each incubated culture was collected and centrifuged at 10,000 x g for 5 minutes. The cell pellet was stored at -20 ^o^ C until thawing for DNA extraction.

*DNA Extraction and Sequencing*

Pellets were extracted following the Zymo DNA Microprep Kit #D4301 (Zymo Research Corporation, USA), with modifications as done previously (Fricker et al. 2024). For 16s rRNA gene sequencing, the variable region 4 of bacterial and archaeal 16S ribosomal RNA genes was amplified based on the original Earth Microbiome Project protocol ((Caporaso et al. 2011); “16S Illumina Amplicon Protocol : Earthmicrobiome” n.d.) with a degenerate 515F and barcoding on the 806R primer. After quantification and equimolar pooling as previously described, samples were sequenced using an Illumina MiSeq (v2, 2 × 150 bp) instrument (Illumina, USA).

*DNA Sequence Analysis*

16S rRNA gene sequences were analyzed following the QIIME-2 Atacama Desert pipeline (Qiime2Docs), and described previously (Fricker et al. 2024). Briefly, sequences were denoised, dereplicated, and chimeras were removed with Dada-2 (Callahan et al. 2016) with the following parameters: --p-trim-left 5 --p-trunc-len 150. Amplicon sequence variants (ASVs) were classified using sklearn and the silva 138.99 database (Quast et al. 2012) . PCR blanks were used to remove contaminating ASVs following the decontamR pipeline (Davis et al. 2018) using a prevalence-based strategy. QIIME-2 was used to calculate various α-diversity (total ASV counts) and β-diversity (Bray Curtis and Jaccard) metrics.

*pH and OD Testing*

Daily, after sampling and subculture, tubes were removed from the anaerobic chamber and opened aerobically. Cultures were transferred to a 15 mL conical vial and 100 μL of the culture was used to determine OD600 nm on a Biophotometer plus spectrophotometer (Eppendorf, Germany). Cultures with an OD600 nm > 1.0 were diluted with sterile water and re-read. The pH was tested on the remaining volume using an accumet pH meter (Fisher Scientific, USA).

*Statistical Analysis*

For pH, optical density, and alpha diversity analyses, statistically significant differences were calculated by Tukey's honest significance multiple comparisons test using R 4.1.1 (R Foundation for Statistical Computing, Vienna, Austria). Comparisons were considered significant if corrected P-values were less than 0.05. For beta-diversity metrics, statistically significant differences between days for each respective donor were calculated by PERMANOVA with 999 permutations using the pairwiseAdonis package (v.0.4.1) in R. Comparisons were considered significant if P-values were less than 0.01.

*Individual Taxa Abundances*

For relative abundances across Day 5 revival and Day 10 development, statistically significant differences were calculated by MaAsLin2 and Spearman's Rank Correlation Coefficient in R 4.5.1. Comparisons were considered significant if the corrected p-values were less than 0.05. Relative abundances of genera that were significantly different across two or more donors were compared using speedyseq and ggtree_3.16.3 after normalizing relative abundances. A color palette ranging from cornsilk (low abundance) to blue (high abundance) was applied to represent abundances between the minimum (0.0) and maximum values (12.5). To illustrate trends of relative abundance across time, line graphs were generated using ggplot2_3.5.2. Error bars represent the Standard Deviation (SD) across lineages.
